# Endogenous analgesic action of the pontospinal noradrenergic system spatially restricts and temporally delays the progression of neuropathic pain following tibial nerve injury

**DOI:** 10.1016/j.pain.2013.05.010

**Published:** 2013-09

**Authors:** S.W. Hughes, L. Hickey, R.P. Hulse, B.M. Lumb, A.E. Pickering

**Affiliations:** School of Physiology & Pharmacology, University of Bristol, Medical Sciences Building, University Walk, Bristol BS8 1TD, UK

**Keywords:** Neuropathic pain, Complex regional pain syndrome, Descending control, Norepinephrine

## Abstract

Pontospinal noradrenergic neurons form part of an endogenous analgesic system that suppresses acute pain, but there is conflicting evidence about its role in neuropathic pain. We investigated the chronology of descending noradrenergic control during the development of a neuropathic pain phenotype in rats following tibial nerve transection (TNT). A lumbar intrathecal cannula was implanted at the time of nerve injury allowing administration of selective α-adrenoceptor (α-AR) antagonists to sequentially assay their effects upon the expression of allodynia and hyperalgesia. Following TNT animals progressively developed mechanical and cold allodynia (by day 10) and subsequently heat hypersensitivity (day 17). Blockade of α_2_-AR with intrathecal yohimbine (30 μg) revealed earlier ipsilateral sensitization of all modalities while prazosin (30 μg, α_1_-AR) was without effect. Established allodynia (by day 21) was partly reversed by the re-uptake inhibitor reboxetine (5 μg, i.t.) but yohimbine no longer had any sensitising effect. This loss of effect coincided with a reduction in the descending noradrenergic innervation of the ipsilateral lumbar dorsal horn. Yohimbine reversibly unmasked contralateral hindlimb allodynia and hyperalgesia of all modalities and increased dorsal horn c-*fos* expression to an innocuous brush stimulus. Contralateral thermal hyperalgesia was also reversibly uncovered by yohimbine administration in a contact heat ramp paradigm in anaesthetised TNT rats. Following TNT there is an engagement of inhibitory α_2_-AR-mediated noradrenergic tone which completely masks contralateral and transiently suppresses the development of ipsilateral sensitization. This endogenous analgesic system plays a key role in shaping the spatial and temporal expression of the neuropathic pain phenotype after nerve injury.

## Summary

A role for the pontospinal noradrenergic system to dynamically restrict the spatiotemporal expression of the neuropathic pain phenotype in a nerve injury model.

## 1 Introduction

Neuropathic pain arises as a direct consequence of a lesion or disease affecting the somatosensory system [55] and is associated with considerable suffering, disability, and impaired quality of life. An estimated 7–8% of the population suffer from neuropathic pain [8], [54] and the condition is poorly responsive to current treatments. Monoamine re-uptake inhibitors (eg tricyclic antidepressants) are one of the more commonly deployed treatments [2] with the most beneficial effects arising from Noradrenaline and (NA) reuptake inhibition [3], [48]. This is suggested to potentiate the actions of a descending pain control system mediated by pontospinal NAergic neurons which provide the sole source of NA in the spinal dorsal horn [36], [44].

These pontospinal projections release NA which acts via inhibitory α_2_-AR on both primary nociceptive afferents and second-order projection neurons to suppress transmission of nociceptive signals [44], [66]. This descending NAergic system plays an important role in acute pain processing [24], [37], [61], [65] and in stress-induced analgesia [7]. Using a targeted retrograde viral vector approach we have shown the restraining effects these neurons have on acute thermal and inflammatory nociception *in vivo* [21].

In contrast, the role of the NAergic system during neuropathic pain has been more difficult to mechanistically define. Several models of neuropathic pain have associated plastic changes within the NA system [4], [16], [50] and there is some evidence to suggest a functional upregulation [31]. Such increased NA tone has been suggested to suppress the expression of neuropathic phenotypes accounting for the “failure rate” in the induction of allodynia and hyperalgesia following nerve injury [12], [62]. However, conflicting evidence exists supporting a functional deficit in the system [45], [58]. Once neuropathic pain is established, previous attempts to uncover a phenotype by blocking descending NAergic control using viral vector-based approaches have been inconclusive [21] as have lesion studies using selective NAergic toxins [22], suggesting that there is little remaining NAergic tone in the system. This proposed functional deficit may account for the inability of the endogenous analgesic system to correct the neuropathic pain phenotype and also explain the clinical and experimental therapeutic benefit from NA re-uptake inhibitors.

These contrasting strands of evidence have led us to examine the chronology of descending NAergic control following nerve injury while neuropathic pain behaviours are developing. Thus we used sequential intrathecal NAergic antagonist/re-uptake inhibitor administration to examine the longitudinal influence of the descending pontospinal NA system on the expression of neuropathic pain in the tibial nerve transection (TNT) variant of the spared nerve injury (SNI) model [28].

## 2 Materials and methods

### 2.1 Animals

Experiments were performed on male Wistar rats (*n* = 59) (Harlan, UK). All procedures conformed to the UK animals (Scientific Procedures) Act 1986. Animals were single housed, with an enriched environment under a standard 12 hours light/dark cycle, with *ad libitum* access to food and water.

### 2.2 Surgery for tibial nerve transection and chronic intrathecal cannulation

**Fig. 1:**
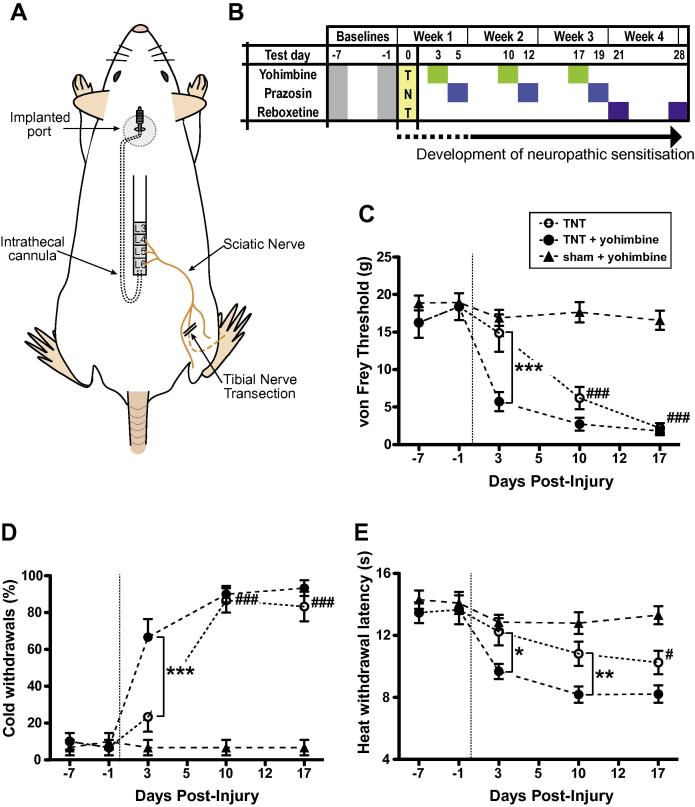
Pontospinal noradrenergic control transiently suppresses ipsilateral allodynia and hyperalgesia. (A) Schematic showing the surgical approach with ligation and tibial nerve transection (TNT) immediately below the trifurcation of the sciatic nerve and insertion of the chronic intrathecal catheter at L5–6 that is exteriorised via an implanted port for subsequent sequential intrathecal dosing. (B) Schedule of the sensory characterisation of the responses to ipsilateral mechanical, cold, and heat stimuli in TNT and sham animals. For each animal the sensory profile for the ipsilateral and contralateral hindlimbs was examined after vehicle (control) and two hours later after active drug administration – either yohimbine (30 μg, i.t.), prazosin (30 μg, i.t.) or reboxetine (5 μg, i.t.). Mechanical allodynia (C) and cold allodynia (D) developed by day 10 in control animals whereas after yohimbine administration allodynia was unmasked at an earlier stage (on day 3). In control animals, heat hyperalgesia was apparent on Hargreaves’ testing by day 17 (E) however yohimbine administration revealed latent heat hyperalgesia to be present at day 3 and day 10. Data are expressed as mean ± SEM, *n* = 6 in each group. Comparisons between vehicle and yohimbine in TNT animals by two-way ANOVA with Bonferroni post tests; ^∗^*P* < 0.01, ^∗∗^*P* < 0.01, ^∗∗∗^*P* < 0.001. Comparisons against baseline values, indicating time of onset of sensitisation following TNT, by one-way ANOVA with Dunnett’s multiple comparison test – significance indicated with ^#^*P* < 0.05, ^##^*P* < 0.01, and ^###^*P* < 0.001.

We used the tibial nerve transection (TNT) variant of the SNI model [28]. Under ketamine (50 mg/kg) and medetomidine (300 μg/kg) anaesthesia, the left hind limb was elevated and secured in a lateral position and an incision was made at the mid-thigh level longitudinally through the biceps femoris. The sciatic nerve was exposed and the sural, tibial, and common peroneal branches were identified. The tibial nerve was then tightly ligated with 5-0 silk and a 2-mm section was cut, avoiding damage to sural and common peroneal nerves (shown schematically in Fig. 1A). Sham surgery consisted of the same procedure without tibial nerve ligation/section.

While still anaesthetised a chronic intrathecal catheter was implanted at the L5–L6 interspace [51], [57]. A sterilised 32-gauge intrathecal catheter (CR3212; ReCathCo; Allison park; PA) was threaded into a 25-gauge hypodermic needle which was inserted between L5 and L6 vertebrae until a tail flick indicated penetration of the dura. The catheter was advanced cranially 2–3 cm so the rostral tip reached the lumbar enlargement. The needle and catheter stylet were removed and the catheter was joined to an 8 cm length of PE-10 tubing which was sutured to the paraspinous muscle and tunnelled subcutaneously to the level of the scapulae. The catheter system was externalised by attaching the PE-10 tubing to a 2 cm length of PE-50 tubing which was fixed to a back mounted pedestal system with a screw cap (313-000BM-10-SP with 6 mm side connector; Plastics One, Roanoak, USA). Animals showing signs of poor health or neurological dysfunction outside the nerve injury territory were excluded from the study (*n* = 3). Correct cannula placement was indicated by immediate and reversible hindlimb paralysis following a 20 μL intrathecal lidocaine injection (10 mg/mL) on the day of surgery.

### 2.3 Nociceptive testing

**Fig. 4:**
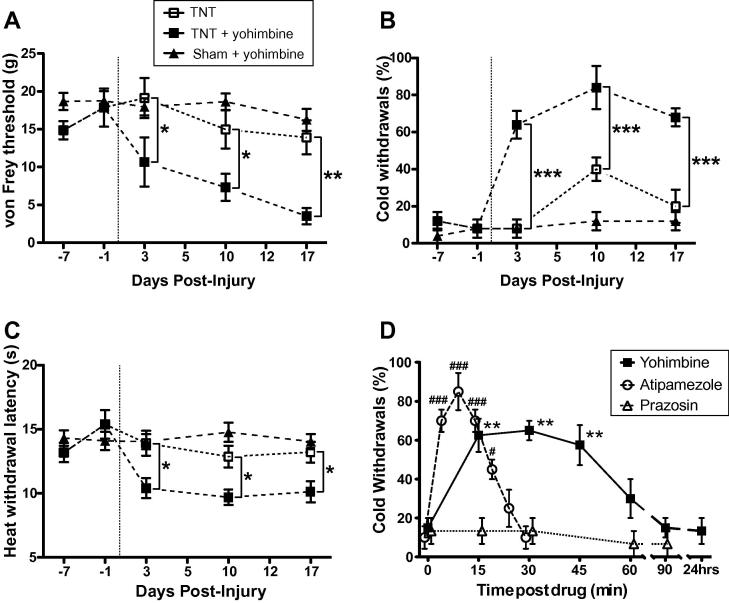
Pontospinal noradrenergic control completely masks contralateral allodynia and hyperalgesia. Sensory testing of the contralateral hindlimb of tibial nerve transection (TNT) animals (compared to sham) showed no significant difference in the response to mechanical, cold, and heat stimuli at any time point (A, B, C; *n* = 6 per group). However the same group of TNT animals tested thirty minutes after yohimbine (30 μg, i.t.) administration developed clear contralateral mechanical allodynia (A) cold allodynia (B) and heat hyperalgesia (C) at all tested time points. (D) This yohimbine (30 μg, i.t.) unmasking of sensitisation was transient peaking between 15 and 45 minutes after dosing and was reversible over the course of an hour (for cold allodynia, *n* = 4). No residual sensitisation was evident after 90 minutes or on testing the following day. This sensitisation was mimicked by administration of the α_2_-AR antagonist atipamezole (50 μg, i.t., *n* = 4) but not by prazosin (*n* = 3) or vehicle (*n* = 4). Data expressed as mean ± SEM. Comparisons between vehicle and yohimbine for TNT animals (A, B, C) by two-way ANOVA with Bonferroni post tests. Comparisons against baseline values, indicating onset of sensitisation, by one-way ANOVA with Dunnett’s multiple comparison test (D) (^∗^*P* < 0.05, ^∗∗^*P* < 0.01, ^∗∗∗^*P* < 0.001).

Behavioural testing was carried out at baseline and at days 3, 5, 10, 12, 17, 19, 21, and 28 post surgery (Fig. 1B). The influence of descending noradrenergic control over time was assayed following single intrathecal doses (10 μL) at sequential time points of either yohimbine (30 μg), prazosin (30 μg), reboxetine (5 μg), or saline. On each study day nociceptive testing was carried out after dosing with saline (control) and again 2 hours later after dosing with the active drug (only 1 drug per day). The nociceptive tests were applied between 15 and 45 minutes after dosing. In time control experiments the effects of drug had completely reversed by the following day (eg see Fig. 4D) and at least 48 hours were allowed to lapse between successive doses.

### 2.4 Punctate mechanical allodynia

The hindpaw withdrawal thresholds to tactile stimuli were assessed using calibrated von Frey filaments ranging from 0.17 to 26.0 g (TouchTest, Linton instruments, UK). Briefly, rats were placed in Perspex chambers with a metal mesh floor and were allowed to habituate for 15 minutes before behavioural testing. Testing started with the 2.0 g von Frey filament, applied perpendicular to the plantar surface of the hindpaw for 3 seconds. Withdrawal thresholds were analysed using the Dixon up/down statistical method [10].

### 2.5 Cold allodynia

Hindpaw withdrawal responses to cooling stimuli were assessed using the acetone test [11]. Following habituation, a 1-mL syringe was used to apply a drop of acetone through the metal mesh floor of the Perspex behavioural chambers to the plantar surface of the hindpaw and a hindlimb withdrawal was scored as a positive response. Acetone testing was repeated 5 times per paw with a 2-minute interval between tests and data are represented as a percentage paw withdrawal frequency (PWF).

### 2.6 Thermal hyperalgesia

The plantar test was used to measure the hindpaw withdrawal latency to heating stimuli [15]. Rats were placed in Perspex chambers and allowed to habituate for 15 minutes. A radiant heat source was focused onto the plantar surface of the hindpaw and latency to withdrawal was recorded (Plantar test, Ugo Basile). A 30-second cut-off time was used to prevent tissue damage and sensitisation.

### 2.7 Dynamic mechanical allodynia

In a further series of experiments the presence of contralateral dynamic mechanical allodynia was sought by brushing the plantar surface of the hindpaw with a thin camel hair brush (continuous for 8 minutes) when animals were at days 19–21 post surgery. Testing was performed on 4 groups of animals: TNT (*n* = 10) or sham (*n* = 6) with half given yohimbine (30 μg, i.t.) and the remainder given vehicle. The animals were sacrificed 2 hours later for trans-cardiac perfuse-fixation with 4% formalin and the lumbar spinal cord removed for c-*fos* immunohistochemistry (see below).

### 2.8 Contact heat ramp-evoked withdrawal

Contact heat ramp-evoked, hindpaw withdrawal experiments were conducted on TNT animals with established allodynia and hyperalgesia (at days 19–21 post surgery) and sham animals (*n* = 20). Anaesthesia was induced using (1–2%) isoflurane in O_2_ until loss of paw withdrawal reflex and the external jugular vein was cannulated for anaesthetic maintenance using continuous intravenous infusion of alphaxalone (5 mg/mL, 9–15 mg/kg/h, Alfaxan; Vetoquinol, UK) and the isoflurane was discontinued. Body temperature was maintained within physiological limits (∼37.0°C) using a feedback controlled heating blanket and rectal probe.

The right carotid artery was cannulated for recording of blood pressure and the trachea was cannulated to maintain a patent airway. A 32-gauge intrathecal catheter (CR3212; ReCathCo; Allison Park; PA) was inserted through a 25-G needle at the L5–L6 interspace and fed rostrally to the lumbar enlargement to allow for drug injection. Bipolar intramuscular electromyogram (EMG) electrodes were inserted into either the ipsi- or contralateral biceps femoris (stainless steel wire, 0.075 mm; Teflon coated, Advent Research Materials, Eynsham, UK).

At the end of this preparatory surgery anaesthesia was lightened by decreasing the infusion rate of Alfaxan (∼10 mg/kg/h) to a level at which animals were moderately responsive to brushing of the cornea using a cotton swab. Animals were allowed to stabilise at their new anaesthetic level for 60 minutes after surgical preparation before recording EMG activity.

The EMG signal was amplified (×5 k) and filtered (50 Hz to 5 kHz; Neurolog; NL104 and NL125), before being captured for analysis (10 kHz) via a 1401plus (Cambridge Electronic Design, Cambridge, UK) onto a PC running Spike2 version 5 software (CED).

Controlled heat ramp stimuli were delivered to the dorsal surface of the hindpaw using a custom-made contact heating lamp assembly (as previously described [35]). The voltage applied to the bulb was adjusted to deliver a heat ramp with a skin surface heating rate of (7.5 ± 1°C/s) monitored from a surface thermocouple. Heat ramps were performed at 8-minute intervals to avoid sensitisation of the hindpaw and a thermal cut off temperature of 58°C was used to prevent tissue damage. The threshold temperature for onset of the EMG withdrawal response was measured for each trial before and after drug dosing.

### 2.9 Drugs

The drugs used in these experiments were yohimbine (α_2_-AR antagonist, 30 μg in 10 μL of 20% DMSO; Tocris, UK), prazosin (α_1_-AR antagonist, 30 μg in 10 μL of 30% DMSO; Tocris, UK; [12], [49], [53], atipamezole (α_2_-AR antagonist, 50 μg in 10 μL saline; Tocris, UK; [62]), clonidine (α_2_-AR agonist, 15 μg in 10 μL of saline, Sigma, UK; [42]), and reboxetine (NA re-uptake inhibitor, 5 μg in 10 μL saline; Tocris, UK; [40]). All intrathecal drug injections were made using a 50-μL Hamilton syringe at a rate of ∼0.5 μL/s followed by a 17-μL dead space flush with saline. Control experiments with intrathecal administration of excipient were without effect on any of the measures of nociception.

### 2.10 Immunohistochemistry

Rats were perfuse-fixed with 4% formalin at days 19–21 post surgery and the spinal cord was cryoprotected in 30% sucrose. Transverse lumbar spinal cord sections (40 μm) were cut from the lumbar enlargement into 3 series using a freezing microtome. For immunocytochemistry they were washed (×3) and permeabilised with 0.1% Triton-X100 in 0.01M phosphate buffered saline (PBS-T). Tissues from all groups were processed together under identical conditions with the same reagents. Controls were routinely run by omission of either primary or secondary antibodies.

To reveal c-*fos* immunoreactivity the sections were incubated free floating with a polyclonal rabbit c-*fos* antibody (SC-52, Santa Cruz Biotechnology; 1:5000 in 0.1M phosphate buffer containing 1% bovine serum albumin, 1% normal goat serum, and 0.1% triton X-100) for 24 hours at room temperature. After further washing (×3) this was followed by incubation with a biotinylated anti-rabbit IgG secondary antibody (Sigma; 1:500 in PBS-T) for 1–2 hours. The sections were then incubated in extravidin peroxidase (Sigma; 1:1000 in PBS-T) for 1–2 hours and the peroxidase visualised using 3,3-diamino-benzidine (0.015%; Sigma) and glucose oxidase (after [20], [27]). The c-*fos*-labelled neuronal profiles were quantified by manually counting in the superficial laminae (I–II) of the dorsal horn (identified under darkfield illumination). The number of c-*fos*-ir profiles were tallied from 3 non-contiguous spinal cord sections from each segment.

Dopamine-β-hydroxylase (DBH) immunohistochemistry was performed on transverse sections (L4–L6) from TNT and sham animals to a similar protocol using a mouse anti-DBH primary antibody (1:5000, Millipore (Chemicon), MAB308) for 24 hours followed by incubation with a biotinylated anti-mouse IgG (Sigma; 1:500 in PBS-T) for 4 hours. The labelling was revealed using the DAB glucose oxidase method as described above [27]. DBH immunoreactivity was quantified for 3 non-contiguous spinal cord sections selected at random from each segment (L4–L6). The mounted sections were examined under brightfield illumination at ×20 magnification (Axioskop 2, Zeiss). Images of the dorsal horns were captured using a charge coupled device camera (Axiocam 3, Zeiss) with the same exposure and illumination setting for all sections. The images were analysed using ImageJ (NIH) to identify the percentage of DBH positive pixels in the grey matter of the dorsal horn. The background level of staining was determined for each section (using ROI analysis of an area without visible DBH fibres) and a value of 5 standard deviations above the mean background level was used to set a threshold level for DBH positive pixels. Each image was manually checked for accuracy and to avoid inclusion of artefactual staining (particularly at the margins of the tissue). The percentage of DBH-positive pixels for ipsi- and contralateral dorsal horns was averaged from 6 sections per animal.

### 2.11 Statistical analysis

Data are presented as mean ± SEM. Differences across groups were determined using either one- or two-way ANOVA or paired/unpaired t-test as appropriate using GraphPad Prism software (GraphPad Software Inc., USA). Levels of significance were set as ^∗^*P* < 0.05, ^∗∗^*P* < 0.01, and ^∗∗∗^*P* < 0.001 (ns, not significant).

## 3 Results

The TNT animals progressively developed robust mechanical and cold allodynia by day 10, with heat hyperalgesia developing later, by day 17 (Fig. 1C–E), which is in line with previous findings using this model [19], [28].

### 3.1 Descending noradrenergic tone transiently suppresses ipsilateral neuropathic sensitisation

Administration of intrathecal yohimbine (30 μg) revealed hindlimb sensitisation at an earlier time point of 3 days for mechanical allodynia (control: 14.8 ± 2.5 g vs yohimbine: 5.7 ± 1.3 g, *P* < 0.001 Fig. 1C); cold allodynia (control: 28 ± 8% vs yohimbine: 72 ± 10% paw withdrawals, *P* < 0.001 Fig. 1D) and heat hyperalgesia (control: 13.5 ± 0.8 s vs yohimbine: 9.5 ± 0.5 s, *P* < 0.05 Fig. 1E). Similar effects were also seen with yohimbine at day 10 for heat hyperalgesia.

**Fig. 2:**
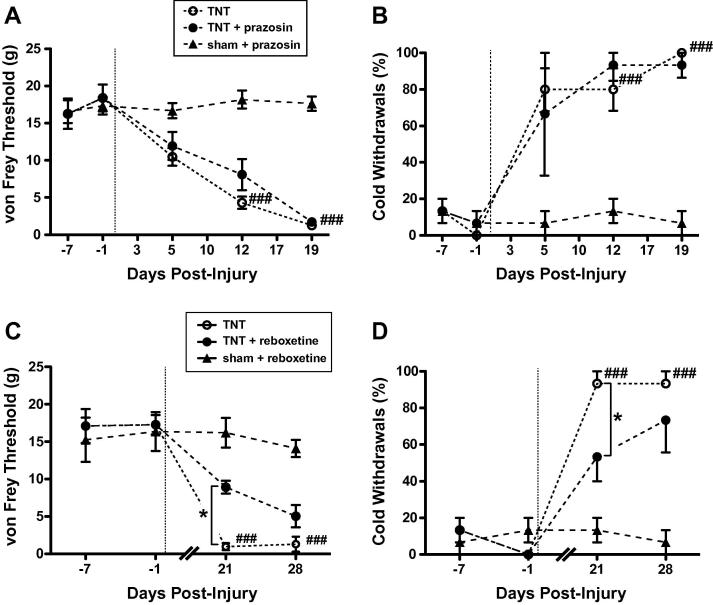
Prazosin has no effect on the development of sensitisation following tibial nerve transection (TNT) whereas reboxetine ameliorates established allodynia. Prazosin administration (30 μg, i.t.) was without significant effect on the development and expression of (A) mechanical allodynia or (B) cold allodynia at any time point. The selective noradrenergic re-uptake inhibitor reboxetine (5 μg, i.t.) ameliorated the signs of established neuropathic sensitisation (3 weeks following nerve injury) and reduced both mechanical (C) and cold allodynia (D). Data are expressed as mean ± SEM, *n* = 5 in each group. Comparisons between vehicle and active drug for TNT animals by two-way ANOVA with Bonferroni post tests; ^∗^*P* < 0.05. Comparisons against baseline values, indicating time of onset of sensitisation following TNT, by one-way ANOVA with Dunnett’s multiple comparison test – significance indicated with ^#^*P* < 0.05, ^##^*P* < 0.01, and ^###^*P* < 0.001.

In contrast intrathecal administration of prazosin (30 μg) had no effect on the development of the neuropathic phenotype at any time point (Fig. 2A,B). Yohimbine and prazosin were without effect on mechanical or thermal sensitivity in sham animals.

Once hyperalgesia and allodynia were established yohimbine was without effect on the mechanical and cold allodynia (day 17). However, this established mechanical and cold allodynia could still be partially reversed by the NA re-uptake inhibitor reboxetine (5 μg, i.t.) which increased paw withdrawal thresholds to mechanical stimuli (*P* < 0.05 at day 21 Fig. 2C) and attenuated paw withdrawals to cold stimulus (*P* < 0.05 at day 21 Fig. 2D). Reboxetine had no effect on established heat hyperalgesia (data not shown).

### 3.2 Tibial nerve transection decreases the descending noradrenergic innervation of the ipsilateral lumbar dorsal horn

**Fig. 3:**
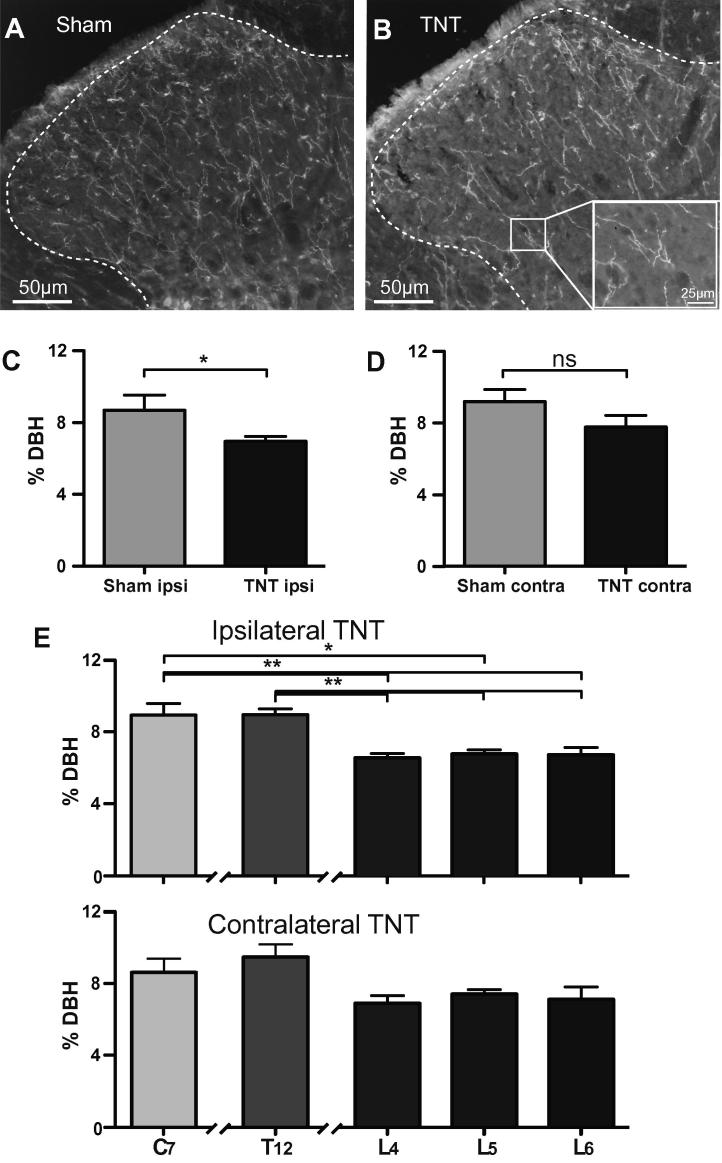
Loss of noradrenergic fibres in the ipsilateral lumbar dorsal horn after tibial nerve transection (TNT). (A) Fewer dopamine-β-hydroxylase (DBH)-ir-positive fibres are seen (white) in transverse spinal cord sections (L6) from the lumbar region in tibial nerve transection (B) animals than in sham operated controls. Inverted brightfield images shown for clarity, (inset shows DBH-ir fibres arrowed). (C) There was a lower density of DBH-ir ipsilaterally in the L6 lumbar dorsal horn in TNT (*n* = 5) compared to sham (*n* = 3) operated rats (unpaired t-test; ^∗^*P* = 0.04, *n* = 6). (D) No significant difference in DBH-ir density on the contralateral side in TNT compared to sham. (E) There was a lower density of DBH-ir in the ipsilateral but not contralateral lumbar segments (L4, L5, L6) compared to cervical (C7) and thoracic (T12) dorsal horns in TNT rats (one-way ANOVA with Dunnet’s multiple comparison test, ^∗^*P* < 0.05, ^∗∗^*P* < 0.01, *n* = 3). Data are expressed as mean ± SEM.

After TNT when the neuropathic sensitisation was established (days 19–21) we found that the density of DBH-ir fibres in the lumbar dorsal horn was significantly lower on the ipsilateral side of TNT animals compared to sham animals (*P* = 0.04, Fig. 3A, B, and C, *n* = 3) whereas there was no significant difference on the contralateral side (Fig. 3D). This loss of fibres appeared contained to the ipsilateral lumbar dorsal horn which had significantly lower density of DBH-ir at L4, L5, and L6 (Fig. 3E, *P* < 0.05, *n* = 3) than cervical (C7) and thoracic (T12) segments. There was no significant loss of contralateral lumbar DBH-ir in TNT animals when comparing across the segments or between sham animals, although in both cases the absolute density of DBH-ir tended to be lower.

### 3.3 Descending noradrenergic tone completely masks contralateral neuropathic sensitisation

**Fig. 5:**
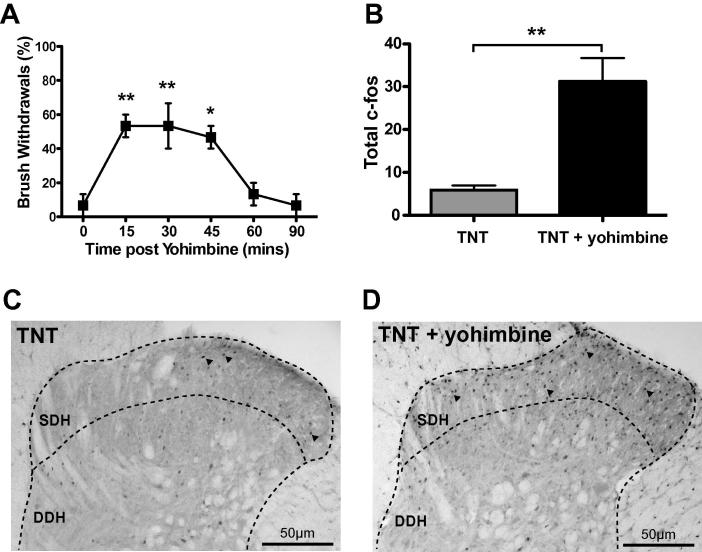
Yohimbine unmasks contralateral dynamic mechanical allodynia and increases superficial lumbar dorsal horn c-*fos* expression in tibial nerve transection (TNT) rats. (A) Time course of contralateral dynamic mechanical allodynia evoked by intrathecal yohimbine (30 μg). The proportion of brush evoked withdrawals (per 5 brush tests, *n* = 3) was significantly and transiently increased for around an hour. (B) Repeated brush stimulation (for 8 minutes) after yohimbine administration greatly increased the expression of c-*fos* in the superficial dorsal horn at L5 compared to control TNT animals (*n* = 5 per group, unpaired t-test; ^∗∗^*P* < 0.01). Shown below in representative sections from (C) control TNT and (D) TNT + yohimbine animals (arrow heads mark c-*fos*-positive nuclei).

TNT animals showed no sensitisation of their contralateral hindlimb responses to nociceptive stimuli over the test period (Fig. 4). However, contralateral mechanical allodynia was revealed by intrathecal injection of yohimbine (30 μg, i.t.) in the same animals from day 3 following TNT (*P* < 0.05, *n* = 6, Fig. 4A) which became more pronounced over time with the lowest thresholds seen by day 17 (13.9 ± 2.2 g to 3.5 ± 1.1 g after yohimbine, *P* < 0.01, Fig. 3A). Yohimbine also unmasked robust contralateral cold allodynia with the proportion of acetone applications producing a paw withdrawal increasing from 8 ± 4% to 64 ± 8% (day 3, *P* < 0.001 Fig. 4B) and contralateral heat hyperalgesia from day 3 (withdrawal latency 13.9 ± 1.0 s at baseline vs 10.4 ± 0.8 s, *P* < 0.05, Fig. 4C). This yohimbine unmasking of sensitisation was maximal between 15 and 45 minutes after administration and reversed over a time course of around an hour (cold allodynia (Fig. 4D, *n* = 4) and dynamic brush allodynia (Fig. 5A, *n* = 3)). This sensitising effect was mimicked by another α_2_-AR antagonist, atipamezole (50 μg, i.t.) which had a shorter duration of action (Fig. 4D, *n* = 4). There was no sign of residual sensitisation on testing the following day (Fig. 4D). Prazosin (30 μg, i.t.) did not change the contralateral responses to nociceptive testing at any time point in TNT animals (see Fig. 4D, *n* = 3) nor did the vehicle control (data not shown, *n* = 4). Yohimbine had no sensitising effect in naïve or sham operated animals.

### 3.4 Spinal α_2_-AR antagonism unmasks contralateral dynamic mechanical allodynia associated with increased dorsal horn c-*fos* expression

Previous studies using the spared nerve injury model have shown innocuous brush stimulation of the ipsilateral hindpaw to trigger withdrawals and evoke increased c-*fos* expression in the superficial dorsal horn - indicating the presence of dynamic allodynia [6]. Having noted the presence of contralateral dynamic mechanical allodynia after yohimbine administration in the TNT model (Fig. 5A). We followed a similar protocol to Bester et al. [6] and after a period of repeated brush stimulation (8 minutes) of the contralateral hindpaw we found a 6-fold increase (*P* = 0.004, *n* = 5, Fig. 5B) in c-*fos*-labelled profiles in the superficial laminae of the L5 dorsal horn in the intrathecal yohimbine group. In sham animals yohimbine administration did not cause an increase in L5 c-*fos* expression to brush stimulus (6±2 vs 7±3), *n* = 3/group). There was a similar level of L5 c-*fos* expression in the sham groups to that seen in the TNT without yohimbine group.

### 3.5 Contralateral hypersensitivity to a ramped thermal stimulus is unmasked by spinal α_2_-AR antagonism

**Fig. 6:**
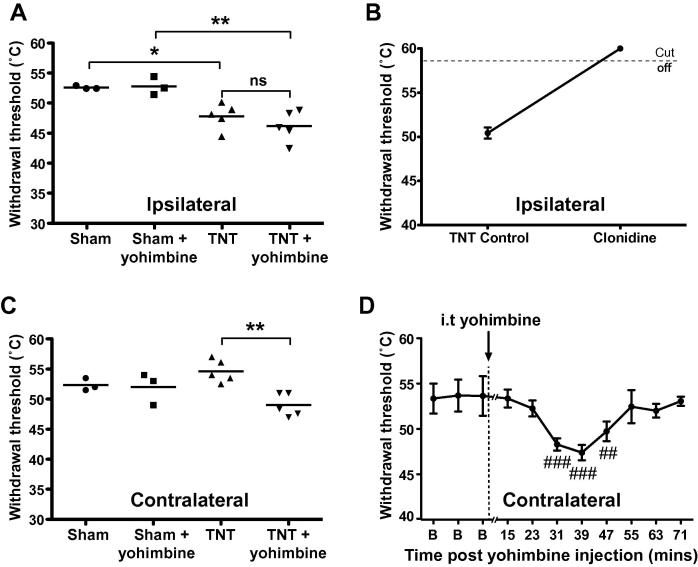
Yohimbine unmasks contralateral hypersensitivity to a ramped heat stimulus. In anaesthetised TNT and sham rats (19–21 days post surgery) the thermal withdrawal threshold to a ramped (7.5°C/s) contact heat stimulus delivered to the ipsilateral or contralateral hindpaw was assayed before and after yohimbine (30 μg, i.t.). (A) TNT animals showed ipsilateral heat hyperalgesia (lower heat withdrawal thresholds) compared to sham (one-way ANOVA with Bonferroni post test; ^∗^*P* < 0.05, ^∗∗^*P* < 0.01). This ipsilateral sensitisation was not altered by intrathecal yohimbine. (B) The ipsilateral heat sensitivity in TNT animals was completely reversed by intrathecal α_2_-AR agonist clonidine (15 μg, *n* = 3) and withdrawal responses were no longer elicited below the cut off threshold (58°C). (C) TNT animals showed similar contralateral heat withdrawal thresholds to sham. Yohimbine unmasked contralateral heat hypersensitivity with significantly lowered thresholds (one-way ANOVA with Bonferroni post test; ^∗∗^*P* < 0.01). (D) Time course of yohimbine unmasking of contralateral heat hypersensitivity showing onset and reversibility over 60 minutes (*n* = 4, one-way ANOVA with Tukey multiple comparison post test; ^##^*P* < 0.01, ^###^*P* < 0.001).

We further tested the influence of the descending noradrenergic system on thermal sensitivity by looking at changes in EMG withdrawal thresholds to a controlled ramped heating stimulus applied to the dorsum of the hindpaw in anaesthetised animals (at days 19–21 post TNT). The TNT animals showed ipsilateral heat sensitisation with lower withdrawal thresholds (47.9 ± 1.0°C TNT vs 52.7 ± 0.2°C in sham, *P* < 0.05 Fig. 6A). Following intrathecal yohimbine there was no change in the ipsilateral withdrawal threshold in either group. However this sensitisation could be reversed by intrathecal clonidine (15 μg, *n* = 3, Fig. 6B) indicating that α_2_-AR receptor function was preserved within these spinal nociceptive circuits.

Contralateral withdrawal thresholds in TNT animals were not sensitised; however yohimbine administration produced a repeatable and reversible sensitisation to the heat ramp stimulus, with a peak change from baseline (54.6 ± 0.8°C vs 47.4 ± 0.6°C, *P* < 0.001, Fig. 6C). The time course of this sensitising effect of yohimbine peaked at around 40 minutes and reversed after approximately 1 hour (Fig. 6D).

## 4 Discussion

We investigated the chronology of influence of the descending NAergic system on the development of the neuropathic pain phenotype following tibial nerve injury. Using a subtractive, longitudinal, intrathecal antagonist approach we have shown that descending NAergic tone delays the appearance of ipsilateral mechanical allodynia, cold allodynia, and heat hyperalgesia following nerve injury via an α_2_-AR mediated mechanism. Once neuropathic sensitisation was established there was no longer any demonstrable effect of α_2_-AR antagonism, suggesting a diminution of the influence of NAergic tone which was echoed anatomically by a diminished density of DBH-ir-positive fibres in the ipsilateral dorsal horn. An unexpected and notable consequence of intrathecal α_2_-AR blockade was the reversible unmasking of pronounced contralateral neuropathic sensitisation to both thermal and mechanical stimulation. These findings indicate that the descending NAergic system acts dynamically to spatially restrict and temporally delay the expression of neuropathic pain at the spinal level. They may also provide insight into the variability of expression of neuropathic sensitisation across animal models and also between patients.

The role of the descending NAergic system in neuropathic pain has attracted considerable attention as NAergic re-uptake inhibitors have been found to be amongst the most effective treatments [2], [48]. However, previous investigations have yielded conflicting evidence regarding the functional role of the system in neuropathic pain. The role of NAergic control has typically been investigated once the neuropathic phenotype is established [21], [22], [31], [45], [46], [58], [62], [63]. At this point subtractive interventions such as pharmacological blockade [21], genetic inhibition or toxin-mediated ablation [17], [21], [23] of the descending NAergic pathway have generally shown minimal or no effect upon sensitisation. Similarly at a cellular level there was no change in response properties of dorsal horn neurons following intrathecal delivery of NAergic antagonists [45]. This led Jasmin et al. [23] to query the hypothesis that tricyclic antidepressants are acting via a NAergic mechanism; however, an alternative explanation is that these subtractive experiments (unlike interventions to facilitate) will only have the power to reveal a phenotype if there is a substantial basal level of tone in the NAergic system in neuropathic pain.

Here we show that the descending NAergic system acts to delay the appearance of neuropathic symptoms in the acute phase after nerve injury. The development of the neuropathic phenotype in the TNT model is slower (often taking over a week to manifest) than other nerve injury models (such as SNI [12]), which may reflect an increased recruitment of endogenous analgesic systems in the early stages after TNT. This NAergic influence diminishes to become undetectable using pharmacological antagonists by day 10 – perhaps because of a floor effect where further sensitisation is no longer discernible or possibly reflecting a functional diminution in the tonic action of NAergic inhibition. This latter possibility is reinforced by a decrease in the density of the NAergic innervation of the lumbar dorsal horn. However, there is still a low level of ongoing NAergic influence even in established neuropathic sensitisation as the reuptake blocker reboxetine can partially reverse the sensitisation as has been shown previously for other monoamine reuptake inhibitors [25], [32], [39], [40], [41]. Additionally we demonstrate that the α_2_-AR function in the spinal nociceptive circuits is still intact after TNT as the sensitisation could be reversed with intrathecal clonidine – in agreement with previous studies [63], [67]. These findings indicate that it is reduced pontospinal NAergic control ipsilaterally that is unable to prevent the expression of sensitisation.

There is a recognised variability in the expression of neuropathic signs following apparently similar surgical injuries in pain models that reflects species, strain, and environmental influences [38]. It has been proposed that one significant factor may be differences in the ability to recruit endogenous analgesic circuits [12], [62]. For example, a subset of animals that failed to exhibit neuropathic signs after spinal nerve ligation (SNL) showed clear evidence of allodynia after intrathecal α_2_-AR blockade suggesting that the NAergic system was acting to oppose sensitisation in this resistant group [62]. A similar observation has recently been made with Holtzman rats that failed to develop allodynia after SNL, where again sensitisation was revealed by spinal α_2_-receptor blockade [12]. This variation may mirror clinical experience where only a minority of patients with apparently similar nerve injuries will go on to develop neuropathic pain [5] and it has been shown that the risk of developing chronic pain after surgery are influenced by the ability to recruit endogenous analgesic systems [64].

There have been reports of upregulation of the NAergic innervation of the spinal cord in nerve injury models, that is chronic constriction injury (CCI) [31] and SNL [16] unlike the segmentally restricted downregulation that we have found here to be associated with functional loss of descending NAergic tone in the TNT model. This may indicate that different NAergic neuroplastic mechanisms are at work in these models of neuropathic pain. Our demonstration of a segmentally localised loss of NAergic innervation (assessed with DBH immunohistochemistry) with sparing of thoracic and cervical segments is consistent with our previous observation of a segmental topography to the LC innervation of the spinal cord [21]. At this point we are unable to discriminate between specific loss of local NAergic fibres or degenerative loss of NAergic neuronal somata or diminished DBH content in the fibres. We note that identification of the underlying mechanism(s) for this NAergic downregulation/retraction may provide a therapeutic target to quell sensitisation in neuropathic pain states.

Blockade of descending NAergic inhibition with intrathecal yohimbine unmasked, from the earliest stages, latent neuropathic sensitisation to all modalities in the contralateral hindlimb of rats with TNT. This yohimbine-induced, transient, contralateral sensitisation was also demonstrated in a contact heat ramp assay – an assay that is known to be subject to modulation by descending control [18], [34]. Similarly, brushing of the contralateral hindpaw after yohimbine triggered paw withdrawals and a markedly increased level of dorsal horn c-*fos* expression consistent with the presence of dynamic mechanical allodynia. None of the TNT animals exhibited significant sensitisation of the contralateral hindlimb without yohimbine blockade, and yohimbine was without effect in sham animals. These findings indicate that the descending NAergic system is dynamically and actively recruited to oppose the expression of neuropathic pain and acts to spatially restrict the sensitised territory via an α_2_-mediated inhibition.

Contralateral neuropathic sensitisation has been reported to occur in some but not all animal models of neuropathic pain including CCI [43], SNL [1] and partial spinal nerve ligation (PSNL) [67]. Variation in the development of contralateral hypersensitivity has also been reported in the SNI model [13], [14]. It has been proposed that such mirror-image sensitisation may be subject to regulation by endogenous analgesic mechanisms [59], and this suppression can be mimicked by the systemic administration of the α_2_-agonist clonidine [67]. We have extended these findings to show that mirror-image sensitisation can be uncovered by blocking descending NAergic tone indicating that these pontospinal neurons are actively opposing the spread of sensitisation at a spinal level. The engagement of such a mechanism to spatially restrict the spread of sensitisation may account for our previous observations of homotopic stimulation-evoked shrinkage of an area of allodynia in patients with neuropathic pain [30].

Clinically, extraterritorial spread of pain following a unilateral nerve injury (ie complex regional pain syndrome (CRPS) type II) is a recognised [56] but confusing and challenging feature of chronic pain states. In particular, mirror pains have long been recognised [29] but within neurological diagnostic sieves where the importance of symptom laterality is emphasised this can be interpreted as indicating “supra-tentorial” psychological factors or the development of a “functional” pain state. The reasons for the bilateral spread of neuropathic symptoms are still unclear but are thought to be partly due to maladaptive neuronal plasticity and/or glial activation at a spinal level [26], [60]. Additionally, there is some evidence to suggest supraspinal changes [33], [52] in CRPS as well as changes in the activity of the patients own endogenous analgesic systems [47]. A key question is why the majority of patients with a unilateral injury do not develop an extraterritorial spread of symptoms – we posit that this may well relate to the ability to recruit descending NAergic control systems that spatially restrict the spread of sensitisation. As such this pontospinal NAergic mechanism provides a potential link between the aforementioned “supra-tentorial” factors and the spinal spread of neuropathic sensitisation.

In summary, the study presented here demonstrates the temporal profile of the endogenous analgesic action of pontospinal noradrenergic neurons during the development of neuropathic sensitisation following a unilateral nerve injury. We have shown that the system transiently inhibits ipsilateral and completely masks contralateral sensitisation; together this indicates that pontospinal NAergic neurons play an important role in shaping the expression of the neuropathic phenotype. We suggest that differences in the susceptibility to developing both ipsilateral and contralateral neuropathic pain seen with nerve injuries across animal models and indeed between individual patients may be in part due to variation in the engagement of the endogenous NAergic analgesic system. Further it may provide a rationale for the early use of NA re-uptake inhibitors in patients with neuropathic pain to delay, ameliorate, and spatially restrict the spread of neuropathic sensitisation – as has been reported to be effective in preventing progression of shingles to post-herpetic neuralgia [9].

## Conflict of interest statement

The authors report no conflict of interest.
